# Supplementary material for: Mapping the evolution of fertility support policies in China: A content and instrumental analysis
Source: PLoS One. 2025 Oct 9;20(10):e0332137. doi: 10.1371/journal.pone.0332137 (PMC12510515; doi:10.1371/journal.pone.0332137)
Supplement: S1 Appendix — (ZIP) [file pone.0332137.s001.zip › S1 Appendix. 226 original policy documents/102-全国农业发展纲要(FBM-CLI-1-129497).doc]

全国农业发展纲要

**发布部门：** [全国人民代表大会](https://www.pkulaw.com/law/chl?Aggs.IssueDepartment=101)

**发文字号：**中华人民共和国主席令2届第15号

**发布日期：**1960.04.11

**实施日期：**1960.04.11

**时效性：** [失效](https://www.pkulaw.com/law/chl?Aggs.TimelinessDic=02)

**效力级别：** [法律](https://www.pkulaw.com/law/chl?Aggs.EffectivenessDic=XA0101)

**法规类别：** [农业管理](https://www.pkulaw.com/law/chl?Aggs.Category=06101)

**失效依据：**[全国人民代表大会常务委员会关于批准法制工作委员会关于对1978年底以前颁布的法律进行清理情况和意见报告的决定](javascript:TMP(3582,0,'chl'))

中华人民共和国主席令
（二届第15号）

　　1956年到1967年全国农业发展纲要已由中华人民共和国第二届全国人民代表大会第二次会议于1960年4月10日通过，现予公布。

中华人民共和国主席　刘少奇
1960年4月11日

全国农业发展纲要
序言

　　这个纲要是在我国第一个到第三个五年计划期间，为着迅速发展农业生产力，以便加强我国社会主义工业化、提高农民以及全体人民生活水平的一个斗争纲领。
　　社会主义工业是我国国民经济的领导力量。但是，发展农业在我国社会主义建设中占有极重大的地位。农业用粮食和原料供应工业，同时，有五亿以上人口的农村，给我国工业提供了世界上的最巨大的国内市场。从这些说来，没有我国的农业，便没有我国的工业。忽视农业方面工作的重要性是完全错误的。
　　发展农业可以有两条道路。一条是资本主义道路：让农民的命运掌握在地主、富农和投机商人的手里，极少数人发财而大多数人贫困和不断破产。一条是社会主义道路：让农民在工人阶级的领导下掌握自己的命运，共同富裕和共同繁荣。这两条道路的斗争在我国过渡时期中将长期地存在，但是，由于农业合作化的基本完成，我国绝大多数农民已经摆脱了前一条道路，走上后一条道路。今后的任务是要尽力巩固合作化制度，同时继续反对农村中的资本主义自发势力。
　　农业合作化给我国农业生产力的发展开辟了最广阔的道路。没有农业合作化，在个体经济的条件下，关于在十二年内在全国几个主要不同地区的粮食，除掉某些例外，争取每亩平均年产量分别达到四百斤、五百斤、八百斤的要求，关于在第二个五年计划时期内争取大多数合作社的生产和收入赶上或者超过当地富裕中农在单干时候的水平的要求，这些都是不可能实现的幻想。但是，在农业合作化以后，加上第一个五年计划中社会主义工业化的伟大成就，经过今后大家千方百计的努力，纲要所提出的这些要求，便有着实现的可能性。
　　由于我国一般的自然条件好，农村劳动力多，农民有勤劳节俭的优良传统和精耕细作的丰富经验，农业经济有很大的潜在力量。必须在合作化的基础上，采取各种积极的合理的措施，并且有准备地有步骤地适合情况地积极推广农业的机械化，充分发掘农业的这种潜在力量，反对保守主义，为着实现纲要的要求而斗争。
　　农业生产水平和农民生活水平的提高，主要依靠农民自己的辛勤劳动。但是，在工人阶级和共产党领导下的人民政府总是尽可能援助农民的。纲要所规定的许多农业增产措施，今后将逐步得到人民政府的更多的必要的援助。在实际上，这是工农的互相支援，城乡的互相支援。
　　以工人阶级为领导的工农联盟和工农互相支援，是农民解放的保证。资产阶级右派分子和封建残余分子为了恢复地主制度和资本主义制度的目的，极力挑拨工农关系和城乡关系。他们这种卑鄙的挑拨失败了，并且还要继续失败下去。
　　要教育农民群众把爱国、爱社和爱家的观念统一起来。没有共产党领导下的中华人民共和国，农民群众就将继续受帝国主义者和地主、富农、投机商人的统治和剥削，就不能有自己的合作社，就将继续出现许多家破人亡的局面。要爱家就得要爱国爱社。一切不顾国家利益和合作社集体利益的本位主义和个人主义，都是错误的，实际结果都将是危害自己家庭利益的。
　　在农业发展的道路上，困难还是会继续出现的。但是，事在人为。对于我们解放了的人民来说，没有什么困难不能克服。不怕困难，是我们劳动人民本来的伟大性格。
　　本纲要是就全国的范围提出的。各地方以至各合作社的情况存在着许多的差别。因此，全国各省（市、自治区）、专区（自治州）、县（自治县）、区、乡（民族乡）的党政领导机关和合作社，都应当根据本纲要，按照本地方、本合作社的具体条件，实事求是，经过群众路线，分别拟定本地方的各项工作的分批分期发展的具体规划。同时，国家各个经济部门，各个科学、文化、教育、卫生部门和政法部门，也都应当根据本纲要，重新审订自己的工作规划。
　　本纲要所述各项任务中，有一些任务，例如绿化，勤俭持家，消灭老鼠、苍蝇、蚊子，消灭危害人民最严重的疾病，提倡有计划地生育子女等，城市居民也应当实行，并且一定要城乡配合进行才能有效地实现。

纲要

　　**（一）**　巩固农业合作化制度

　　我国的农业合作化已经在一九五七年基本完成。今后的任务是：争取在第二个五年计划时间内，或者更多一点时间，把所有的农业生产合作社巩固起来。

　　巩固合作社的条件是：（1）在合作社的领导成分中，保持原来的贫雇农和下中农（主要是现在的贫农和新中农中间的下中农）的优势，同时注意使上中农也有适当的代表。（2）贯彻执行民主办社的方针。合作社领导机关要按时公布财务收支，干部要同群众商量办事，参加生产劳动。（3）贯彻执行勤俭办社的方针。要同一切游手好闲的现象作斗争，反对铺张浪费。（4）根据合作社的经济情况和当地的自然情况，采取各种增产措施，逐步地增加农业基本建设，保证遵守和完成国家的计划，不断地扩大再生产，争取在第二个五年计划时期内，使大多数合作社赶上或者超过当地富裕中农的生产他一切有销路的经济作物。华南各省有条件的地区，应当注意发展热带水平和收入水平。（5）合理地处理分配问题，兼顾国家、合作社、社员三方面的利益，在发展生产和正常年景的情况下，使合作社的公共积累和社员的收入逐年有所增加，争取在一九六二年前后，合作社集体经济的收入，加上社员家庭副业收入，按人口平均，赶上或者超过当地富裕中农的收入。（6）加强政治思想工作，不断地提高社员的社会主义觉悟，克服资本主义思想，克服不顾国家利益和合作社集体利益的本位主义思想和个人主义思想；每年应当结合农村干部的整风和合作社的整社，系统地总结本年度的工作，在全体农村人口中集中地进行一次社会主义教育。在多民族的地区，要特别注意民族间的团结和互助。

　　现有的、数目不多的初级合作社，在条件成熟了的时候，应当引导它们自愿地转为高级合作社。加强对现在还存在的少数个体农民的教育和领导，争取他们自愿地陆续加入合作社；不愿入社的，听其自便。

　　**（二）**　大力提高粮食的产量和其他农作物的产量

　　从一九五六年开始，在十二年内，粮食每亩平均年产量，在黄河、秦岭、白龙江、黄河（青海境内）以北地区，由一九五五年的一百五十多斤增加到四百斤；黄河以南、淮河以北地区，由一九五五年的二百零八斤增加到五百斤；淮河、秦岭、白龙江以南地区，由一九五五年的四百斤增加到八百斤。其中的沙荒地区、土地瘠薄地区、常年旱涝地区、高寒山区、无霜期很短地区、地广人稀地区、大面积垦荒地区，可以按照情况，另外规定增产指标。

　　从一九五六年开始，在十二年内，棉花每亩平均年产量（皮棉），由一九五五年的三十五斤（全国平均数），按照各地情况，分别增加到四十斤、六十斤、八十斤和一百斤。

　　在优先发展粮食生产的条件下，各地应当发展农业的多种经济，保证完成国家所规定的纺织原料（棉花、麻类、蚕茧），油料（大豆、花生、油菜籽、芝麻、油茶、油桐），糖料（甘蔗、甜菜），茶叶，烤烟，果类，药材等项农作物的计划指标，还应当积极地发展其他一切有销路的经济作物。华南各省有条件的地区，应当注意发展热带和亚热带作物。

　　农业合作社应当鼓励社员在自留地上种植蔬菜和饲料。城市郊区和工矿区附近的合作社和国营农场，应当按照国家的计划种植蔬菜，充分地保证城市和工矿区的蔬菜供应。

　　**（三）**　发展畜牧业

　　畜牧业的合作化，应当按照各地情况，分别规定不同的发展计划。有计划地发展国营牧场。大力保护和繁殖牛、马、驴、骡、骆驼、猪、羊、兔等家畜和适当地繁殖各种家禽。特别注意保护母畜、幼畜和种公畜。建立其种站，改良畜种。

　　在牧区要保护草原，改良和培植牧草，特别注意开辟水源。牧业合作社应当逐步建立自己的饲料和饲草的基地。推广青贮饲料。

　　分别在七年或者十二年内，在一切可能的地方，基本上消灭危害牲畜最严重的病疫，例如牛瘟、猪瘟、鸡瘟、疲肺疫、口蹄疫、猪囊虫、羊痘、羊疥癣等。在一九六二年以前，农业区的县或者区和牧业区的区或者乡，应当建立起畜牧兽医工作站。合作社应当有初级的防治兽疫的人员。充分发挥民间兽医人员的力量，组织和领导他们提高技术，参加防治兽疫的工作。

　　**（四）**　推行增产措施和推广先进经验，是增加农作物产量的两个基本条件

　　增产措施的项目，主要是：（1）兴修水利。（2）增加肥料。（3）改良旧式农具和推广新式农具。（4）推广优良品种。（5）扩大复种面积。（6）多种高产作物。（7）实行精耕细作，改进耕作方法。（8）改良土壤。（9）保持水土。（10）保护和繁殖耕畜。（11）消灭虫害和病害。（12）开垦荒地，扩大耕地面积。

　　推广先进经验的方法，主要是：（1）由各省、市、自治区收集当地的丰产经验，编印成书，传播推广。（2）举办农业展览会。（3）各级政府定期召开农业劳动模范会议，奖励丰产模范。（4）组织合作社之间的参观和评比，交流增产经验。（5）在总结先进经验的基础上，组织技术传授，发动农民和干部学习外社外乡外县外省（自治区）的先进的管理经验和技术知识。

　　**（五）**　兴修水利，发展灌溉，防治水旱灾害

　　从一九五六年起，在十二年内，全国水利事业的发展，应当以修建中小型水利工程为主，同时修建必要的可能的大型水利工程。

　　小型水利工程（打井、挖塘、筑堤、打旱井、开渠、筑圩、修水库、兴修蓄水排水的沟洫畦田台田系统等），小河的治理，都由地方和农业合作社负责，有计划地尽可能大量地进行。通过这些工作，结合国家大中型水利工程的建设和大、中河流的治理，要求在十二年内，基本上消灭普通的水灾和旱灾。

　　内涝灾害严重的地区，应该大力进行除涝排水、改造洼地的工程建设。

　　要求在十二年内，把水田和水浇地的面积，由一九五五年的三亿九千多万亩扩大到九亿亩左右。灌溉设施的抗旱能力，按各地不同情况，分别提高到三十天到五十天；适宜发展双季稻的地方，要提高到五十天到七十天，以保证收成。为了充分利用一切水源，有关部门应当积极进行对地下水的勘察工作，保证水利部门所需要的必要资料。

　　凡是能够发电的水利建设，应当尽可能同时进行中小型的水电建设，结合国家大中型的电力工程建设，逐步增加农村用电。

　　**（六）**　大力增加农家肥料和化学肥料

　　农业合作社要采取一切办法，尽可能由自己解决肥料的需要。应当特别注意养猪（有些地方养羊）。除了某些不养猪的少数民族地区和因为宗教习惯不养猪的少数家庭以外，要求一九六二年达到农村平均每户养猪一头半到两头，一九六七年达到农村平均每户养猪二头半到三头。要做到猪羊有圈，牛马有栏。还应当因地制宜地积极发展各种绿肥作物，并且把城乡的粪便，可作肥料的垃圾和其他杂肥尽量利用起来。

　　中央和地方都应当积极发展化学肥料的制造工业，争取到一九六二年生产化学肥料五百万吨到七百万吨左右，一九六七年生产一千五百万吨左右。积极发展细菌肥料。

　　**（七）**　改良旧式农具，推广新式农具

　　根据生产的需要，经过地区适应性的试验和改制，积极推广那些适合当地条件的改良农具和新式农具。经常注意农具的保养和修理的工作。加强新式农具使用的技术指导。

　　随着国家工业化的发展，有步骤地积极地实行农业机械化。从一九五六年起，在尽可能短的时间内，机械制造部门和农业部门应当经过广泛的试验研究工作，拟出一个适合我国条件的农业机械化方案，为推广农业机械化做好准备，随即制造适合各地的机械，供应农民；随时改良，积极推广。

　　**（八）**　积极繁育和推广适合当地条件的农作物优良品种

　　要求在一九六二年以前，经过地区适应性的典型试验，普及各种农作物现有的优良品种。对于良种已经基本上普及的作物（例如棉花），应当加强种子复壮和品种更换的工作。大力培育新的良种，并且注意试种外地和外国的良种。

　　农业合作社应当建立自己的种子地，加强群众的选种工作，建立农作物良种繁育更换制度。在丰歉经常不定的地区，要注意储备优良品种的种子。中央和地方的国营农场应当成为繁育农作物良种的基地，积极繁殖和推广适合当地的农作物良种。各省（市、自治区）、专区（自治州）、县（自治县）都应当建立种子管理机构。

　　**（九）**　扩大复种面积

　　从一九五六年起，在十二年内，按照不同地区的不同条件，把复种指数（包括绿肥作物在内）分别提高到下列的水平：（1）五岭以南地区，达到百分之二百三十左右。（2）五岭以北、长江以南地区，达到百分之二百左右。（3）长江以北，黄河、秦岭、白龙江以南地区，达到百分之一百六十左右。（4）黄河、秦岭、白龙江以北，长城以南地区，达到百分之一百二十左右。（5）长城以北地区，一般应当尽可能地利用已有耕地，减少撩荒面积，在可能的地方，力争扩大复种面积。

　　**（十）**　多种高产作物

　　利用一切可能利用的水源，增加稻谷的种植面积。从一九五六年起，在十二年内，要求增加稻谷二亿五千万亩。根据需要和民食习惯，适当地发展玉米和薯类等高产作物。

　　**（十一）**　积极地因地制宜地改进耕作方法

　　一切农业合作社都应当实行精耕细作，改进耕作方法，合理地施肥，合理地灌溉，合理地输作（换茬）、间作、套种和密植。不违农时，及时播种，及时锄草间苗，加强田间管理，及时收获，细收净打，力求丰产保收。

　　**（十二）**　改良土壤农业

　　合作社和国营农场都应当积极改良和利用盐碱地、瘠薄的红土壤地、低洼地、砂地和其他各种瘠薄的土地。注意防止土地的盐碱化。山地必须有计划地积极地整修梯田。用各种办法把瘠薄的土地变成肥沃的良田好地。

　　**（十三）**　开展保持水土的工作

　　在有水土冲刷的地区，应当依靠农业合作社，广泛地发动群众，结合当地的生产，有步骤有计划地开展保持水土的工作。从一九五六年起，要求在十二年内，在一切可能的地方，显著地收到保持水土的功效，逐步减少水土流失的损害。为使此项工作能够较快地收到效果，农业、林业、水利、牧业和科学研究等有关部门，必须在当地党政机关的统一领导下，密切配合，积极支援。

　　**（十四）**　保护和繁殖耕畜

　　从一九五六年起，要求在七年到十二年的时间内，每一个农业合作社都要有足够数量的强壮的耕畜。为此，必须生产足够的饲草、饲料，种植高产饲料作物。合作社应当根据自己的条件，不断地改进耕畜的饲养管理工作。合作社和政府应当采取正确的措施奖励耕畜的繁殖。

　　**（十五）**　防治和消灭病虫害

　　从一九五六年起，分别在七年或者十二年内，在一切可能的地方，基本上消灭危害农作物最严重的虫害和病害，例如蝗虫、稻螟虫、粘虫、玉米螟虫、棉蚜虫、棉红蜘蛛、棉红铃虫、小麦吸浆虫、麦类黑穗病、小麦线虫病、甘薯黑斑病等；同时防止其他危险性的病害、虫害、杂草的传播蔓延。各地区应当把当地其他可能消灭的主要虫害和病害，列入消灭计划之内。为此，必须加强植物保护工作和植物检疫工作。

　　有计划地发展农药和药械的生产，提高产品质量，改进供应工作。同时，加强使用上的技术指导，保证安全有效。

　　**（十六）**　开垦荒地，扩大耕地面积

　　国家应当有计划地开垦荒地。从一九五六年起，在十二年内，要求国营农场的耕地面积由一九五五年的一千三百多万亩增加到一亿亩左右。在有条件的地方，应当组织移民和鼓励合作社组织分社或者派出生产队，进行垦荒。在垦荒的时候，必须同保持水土和发展畜牧业的规划相结合，避免水土流失，避免破坏树林和破坏必需的草原。在树林已经遭到破坏的地方，应当努力恢复。

　　农业合作社应当充分利用田埂、地角、塘边、沟沿、空闲的场院等一切零星的土地。这些土地可以根据情况，分配给生产队或者社员个人经营。

　　工矿、商业、农业等企业，文教、卫生、水利、交通、军事等部门和群众团体，在进行基本建设的时候，都必须尽量节省用地，尽量避免和减少占用耕地。

　　**（十七）**　发展山区经济

　　按照因地制宜、实行多种经营的方针，发展山区的农业、林业、畜牧业和各种土特产的生产。

　　在不破坏水土保持的原则下，发展山区的粮食生产。现在还缺粮的地区，应当争取作到粮食自给，或者减少粮食调进。

　　远山区，应当着重发展用材林。近山区，应当着重发展特用经济林、薪炭林、各种果木和土特产。南方的山区，应当注意发展油茶、油桐、竹子、桑树、茶树等。北方的山区，应当注意发展核桃、柞树、花椒等。

　　发展药材生产。注意保护野生药材，并且根据可能条件逐步进行部分的人工栽培。

　　从一九五六年起，在十二年内，在一切可能的地方，基本上消灭危害山区生产最严重的兽害。保护和发展有经济价值的野生动物。

　　山区、半山区的专区、县、乡和农业合作社，都应当制定较长期的山区生产规划，合理地使用土地，促进生产的全面发展。国家各有关部门，应当从交通运输、供销贸易、文教卫生、组织移民等方面作有力的配合和支援。

　　**（十八）**　发展林业，绿化一切可能绿化的荒地荒山

　　从一九五六年起，在十二年内，在自然条件许可和人力可能经营的范围内，绿化荒地荒山。在一切宅旁、村旁、路旁、水旁，只要是可能的，都要有计划地种起树来。为此，必须依靠农业合作社造林，实行社种社有的政策。要求合作社自己采集树籽和培育树苗，采取分工负责、包栽包活的办法。同时，鼓励社员在自己的宅旁种树，自种自有。

　　种树，除了用材林（包括竹林）以外，应当尽量利用一切人力和城乡空地，发展果木、桑、柞、茶、漆、油料等经济林木。

　　大力加强国营造林。国营林场以营造大片用材林为主，并且有计划地营造水土保持林、防风林、防沙林和海防林。

　　铁路、干线公路和大河流两旁、大型水库周围、矿山附近的绿化，由铁路、交通、水利部门和厂矿负责经营，收益归各经营部门；也可以由附近的农业合作社经营，按照有关部门所定的规格造林，收益归合作社。

　　绿化用地，不应当占用耕地。

　　要求在十二年内，尽可能地把国有森林全部经营管理起来。国家不便经营管理的小规模的国有林，应当委托合作社经营管理。必须保护和爱惜森林资源，加强防火工作，防治虫害和病害，制止滥伐和采伐当中浪费木材的现象，并且及时更新采伐迹地，恢复森林。

　　**（十九）**　发展海洋和淡水的水产品生产和水产养殖业

　　在海洋渔业中，应当在合作化的基础上，发挥现有捕捞工具的潜力，逐步改进生产技术。应当注意增加公共积累，添置和改良生产工具，逐步发展机帆船和轮船。加强生产的安全措施，争取向深海发展。利用一切可能养鱼的水面，发展淡水养殖业。加强培育优良鱼种和防治鱼瘟的工作。积极发展浅海养殖业，加强鱼类、藻类、贝类的养殖。

　　**（二十）**　办好国营农场

　　要求所有的国营农场，团结和帮助周围的农业合作社，在生产技术方面发挥应有的示范作用。国营农场应当实行多种经营，提高劳动力利用率；不断地改进生产技术和劳动管理，提高劳动生产率。必须厉行节约，贯彻执行勤俭办农场的方针，改善工资制度，既要增加产量，又要降低成本。

　　**（二十一）**　改善农业科学研究工作和技术指导工作

　　根据需要和可能条件，建立和改进农业科学研究工作和技术指导工作的机构，例如农业科学院，区域性的和专业性的农业科学研究所，省的农业试验站，县的示范繁殖农场和农业技术服务站等，使农业科学研究工作更好地为发展农业生产服务。

　　农业科学研究和技术指导，必须同农民群众的生产实践密切地结合起来。必须总结当地农民特别是老农的增产经验，学习其他地方农业生产上的好经验。县的农业技术服务站的工作人员，都应当分别选择重点的农场和农业合作社作为工作的基地，参加农业生产。

　　从一九五六年开始，在十二年内，要从具有相当生产经验和一定文化程度的农民中间，为农业合作社培养初级的和中级的技术人才，以适应合作经济发展的需要。

　　**（二十二）**　加强气象水文工作

　　从一九五六年起，在十二年内，基本上建成气象台站网和水文观测站网。加强天气预报，特别是危险天气预报的工作。建立农业气象预报的工作。各地应当注意收听关于气象的广播，以便预防水、旱、风、冻等自然灾害。

　　**（二十三）**　勤俭办社，勤俭持家

　　一切农业合作社都必须实行勤俭办社的原则。勤，就是要发动社员勤劳生产，在可能和必要条件下积极地扩大生产范围，发展多种经济，进行细致工作。俭，就是要厉行节约，降低生产成本，反对铺张浪费。合作社的各种基本建设，都应当尽量利用合作社本身的人力、物力和财力。

　　应当教育农民勤俭持家，长远打算，有计划地安排各项家务开支。农村中的妇女团体，应当把教育妇女勤俭持家，作好家务劳动，当作自己的一项重要任务。注意介绍农家精打细算、省吃俭用、增加积蓄和积累的模范事例。不作长远打算、不注意家庭积累、随时吃光用光的思想，是错误的。农村中的红白喜事、人情应酬，应当一切从简，改变原来不合理的风俗习惯。

　　**（二十四）**　提高农业合作社的劳动力利用率和劳动生产率，发展多种经济

　　为了充分发展农业和林、牧、渔等副业以及农村手工业，努力扩大生产门路，增加社会财富和农村人民的收入，农业合作社应当提高劳动力的利用率。北方要设法增加冬季生产。同时，还应当积极改进生产技术，改善劳动组织和劳动管理，不断地提高社员的劳动生产率。

　　从一九五六年开始，在七年内，要求做到农村中的每一个男子全劳动力每年至少做二百五十天左右的工作。根据妇女的特点，合理地安排和组织妇女劳动力。妇女除了从事家务劳动的时间以外，在七年内，根据不同地区的不同情况，要求做到每一个农村女子全劳动力每年参加农业和副业（包括家庭副业）生产劳动的时间不少于八十天到一百八十天。此外，对于农村中一切具有半劳动力的人们，或者能够从事轻微劳动的人们，合作社应当作好适当的安排，要求他们积极地分别参加适合自己能力的劳动。

　　**（二十五）**　储备粮食

　　要求一切农业合作社，除了主要是经营山林或者经营经济作物的缺粮社以外， 从一九五六年起，在十二年内，按照自己的情况，定出具体计划，加上社员家庭的粮食储备，分别储积足够三个月、半年、一年或者一年半食用的余粮，以备紧急时候的需要。在丰歉经常不定和交通不便的地区，特别要注意以丰补歉，积谷防荒。

　　从一九五六年起，在十二年内，国家应当储备足够一年到两年之用的粮食，以应急需。

　　**（二十六）**　改善居住条件

　　随着合作社生产的发展和社员收入的增加，农业合作社应当根据需要和可能，鼓励和协助社员，在自愿、互助、节约开支和节省用地的原则下，有准备地、有计划地、分批分期地修缮和新建家庭住宅，改善社员的居住条件。

　　**（二十七）**　除四害

　　从一九五六年起，在十二年内，在一切可能的地方，基本上消灭老鼠、臭虫、苍蝇和蚊子。

　　**（二十八）**　努力消灭危害人民最严重的疾病

　　从一九五六年起，在十二年内，在一切可能的地方，基本上消灭危害人民最严重的疾病，例如：血吸虫病、天花、鼠疫、票疾、黑热病、钩虫病、血丝虫病、新生儿破伤风和性病。其他疾病，例如：麻疹、赤痢、伤寒、流行性乙型脑炎、脊髓灰白质炎、白喉、肺结核、麻疯、沙眼、甲状腺肿、大骨节病、克山病等，也应当积极防治。积极培养医务卫生人员，包括中医在内。

　　积极开展群众的经常性的爱国卫生运动，养成人人讲卫生、家家爱清洁的良好习惯。讲求清洁卫生的根本精神，是为了消灭疾病，人人振奋，移风易俗，改造国家。

　　**（二十九）**　保护妇女儿童

　　对于妇女的生产劳动，坚决实行同工同酬的原则。农业合作社在必要和可能的条件下，可以成立适合需要的临时简便的农忙托儿组织。在分配工作的时候，对于女社员的生理特点应当予以照顾。对于农村儿童参加农忙时期的辅助劳动，应当根据他们的年龄和体力，做出适当的规定。

　　卫生部门应当为农村训练助产员，积极推广新法接生，保护产妇和婴儿，降低产妇的染病率和婴儿的死亡率。

　　除了少数民族的地区以外，在一切人口稠密的地方，宣传和推广节制生育，提倡有计划地生育子女，使家庭避免过重的生活负担，使子女受到较好的教育，并且得到充分就业的机会。

　　**（三十）**　实行“五保”，优待烈属和残废革命军人，供养和尊敬父母

　　农业合作社对于社内缺乏劳动力、生活没有依靠的鳏寡孤独的社员，应当统一筹划，指定生产队或者生产小组在生产上给以适当的安排，使他们能够参加力能胜任的劳动；在生活上给以适当的照顾，做到保吃、保穿、保烧（燃料）、保教（儿童和少年）、保葬，使他们的生养死葬都有指靠。

　　对于缺乏劳动力的烈属和享受残废抚恤金以后仍然不能维持生活的残废革命军人，合作社应当按照国家规定的优待办法给以优待，使他们的生活不致低于一般社员的水平。

　　应当教育青壮年男女供养和尊敬自己的父母，使年老的丧失劳动能力的人，在生活上得到合理的照顾，在精神上得到充分的安慰。

　　**（三十一）**　扫除文盲，发展农村文化教育事业

　　从一九五六年开始，按照各地情况，分别在十二年内，基本上扫除青年和壮年中的文盲。争取在乡或者社逐步设立业余文化学校，以便进一步提高农村基层干部和农民的文化水平。农村办学应当采取多种形式，除了国家办学以外，必须大力提倡群众集体办学，允许私人办学，以便逐步普及小学教育。

　　随着农业生产的发展，合作社应当根据可能的条件，按照勤俭建国、勤俭办社、勤俭持家的原则，逐步改进和开展文化娱乐工作。

　　**（三十二）**　发展农村广播网

　　从一九五六年起，按照各地情况，分别在七年或者十二年内，基本上普及农村广播网。要求大部分农业、林业、渔业、牧业、盐业和手工业的生产合作社都能收听广播。

　　**（三十三）**　发展农村的电话网和邮政网

　　从一九五六年开始，按照各地情况，分别在七年或者十二年内，建立乡和某些合作社的电话网，有些地区可以设置无线报话器。在十二年内，基本上普及农村邮政网，做好邮电传递工作。

　　**（三十四）**　发展农村交通运输

　　从一九五六年起，按照各地情况，分别在七年或者十二年内，基本上建成全国地方道路网。在省（市、自治区）、专区（自治州）、县（自治县）、区、乡（民族乡）之间，根据当地运输发展的需要，按照实事求是、因地制宜、就地取材、经济适用、不浪费土地的原则，依靠群众力量，逐步修建适应当地运输工具的各种道路。同时作好各种道路的经常养护工作。特别要注意山区道路的修建。

　　有河道可通的地方，应当在可能的条件下，整理和疏浚航道，以利交通。

　　**（三十五）**　调整农村商业网

　　为了适应农业合作化以后的新情况，商业部门和农村供销合作社，应当在最近几年内，完成农村商业网的调整，加强商品的流通，加强初级市场的管理，保证做好农村中的商品供应工作和农产品的收购工作，同资本主义的自发势力作斗争，促进农村生产的发展。

　　**（三十六）**　发展农村信贷合作事业

　　巩固农村信用合作社，积极开展农村信贷业务和农村储蓄业务，帮助农业合作社和农民解决短期资金周转的需要。

　　**（三十七）**　发挥复员军人建设社会主义农村的积极性

　　在农村的复员军人应当在社会主义生产战线上保持人民解放军的优良传统，艰苦朴素，联系群众，遵守政策、法令和劳动纪律，成为农业生产中的积极分子。乡人民委员会和农业合作社要适当地安排复员军人的生产活动，帮助他们学习和提高生产技术。各级人民政府要经常关心复员军人的进步，对他们进行政治思想教育。

　　**（三十八）**　提高农村青年的社会主义积极性

　　教育农村青年热爱祖国、热爱农村、热爱劳动和爱社如家，鼓励他们积极地学习文化和农业科学技术，学习老年和壮年农民的生产经验。农村青年应当成为农村的生产建设和科学文化事业中的突击力量。

　　城市的中、小学毕业的青年，除了能够在城市升学、就业的以外，应当积极响应国家的号召，下乡上山去参加农业生产，参加社会主义农业建设的伟大事业。我国人口百分之八十五在农村，农业如果不发展，工业不可能单独发展。到农村去工作是非常必要的和极其光荣的。

　　**（三十九）**　改造地主、富农、农村中的反革命分子和其他坏分子，保护农村的社会主义秩序

　　过去的地主分子、已经放弃剥削的富农分子和农村中的过去的反革命分子，按照“[高级农业生产合作社示范章程](https://www.pkulaw.com/chl/6d0fcab3bc0f13f1bdfb.html#class=alink)”的规定，根据他们的实际表现，可以由农业合作社分别吸收他们入社做社员或者做候补社员。不够入社条件的，可以由乡人民委员会交给合作社监督生产。对于这些人，合作社要分别情况加强教育和加强管理，并且要经常地教育社员和社外农民，提高警惕性，防止他们中间可能发生的破坏活动。已经成为社员的或者候补社员的过去的地主分子、富农分子和反革命分子，如果表现不好，并且屡教不改，是社员的，可以分别降为候补社员或者监督生产；是候补社员的，可以降为监督生产。如果有破坏行为，还应当给予法律制裁。

　　严禁赌博，取缔会道门活动。对于盗窃犯、诈骗犯、流氓分子、特务分子和各种严重破坏社会秩序的坏分子，都必须依法惩办。

　　**（四十）**　城市工人和合作社的农民必须互相支援

　　工人应当生产更多更好的工业品，满足农民的需要；农民应当生产更多更好的粮食和工业原料，满足工业和城市居民的需要。城市的工人和合作社的农民，还应当通过联欢、访问和通信等项方法，加强联系，互相鼓励，交流经验，以便有利于工业和农业的发展，有利于在工人阶级领导之下的工农联盟的巩固。

© 北大法宝：（[www.pkulaw.com](https://www.pkulaw.com/)) 专业提供法律信息、法学知识和法律软件领域各类解决方案。北大法宝为您提供丰富的参考资料，正式引用法规条文时请与标准文本核对。
欢迎查看所有[产品和服务](https://www.pkulaw.com/helps/2.html)。法宝快讯：[如何快速找到您需要的检索结果？](https://www.pkulaw.com/helps/70.html)[法宝](https://www.pkulaw.com/helps/69.html)V6有何新特色？

扫描二维码阅读原文

**原文链接：**<https://www.pkulaw.com/chl/27d45dd503fdd610bdfb.html>
